# CREPE: a Shiny app for transcription factor cataloguing

**DOI:** 10.1093/bioadv/vbad055

**Published:** 2023-04-21

**Authors:** Diego A Rosado-Tristani, José A Rodríguez-Martínez

**Affiliations:** Department of Biology, University of Puerto Rico Río Piedras, San Juan 00925, Puerto Rico; Department of Biology, University of Puerto Rico Río Piedras, San Juan 00925, Puerto Rico

## Abstract

**Summary:**

Transcription factors (TFs) are proteins that directly interpret the genome to regulate gene expression and determine cellular phenotypes. TF identification is a common first step in unraveling gene regulatory networks. We present CREPE, an R Shiny app to catalogue and annotate TFs. CREPE was benchmarked against curated human TF datasets. Next, we use CREPE to explore the TF repertoires of *Heliconius erato* and *Heliconius melpomene* butterflies.

**Availability and implementation:**

CREPE is available as a Shiny app package available at GitHub (github.com/dirostri/CREPE).

**Supplementary information:**

Supplementary data are available at *Bioinformatics Advances* online.

## 1 Introduction

Transcription factors (TFs) are sequence-specific DNA-binding proteins that regulate transcription. TF proteins bind to cis-regulatory elements (CREs), genomic regions that regulate the expression of nearby genes. Examples of CREs include promoters, enhancers and silencers (Wittkopp and Kalay, 2011). By regulating spatiotemporal gene expression, TFs determine cell fate during development and cellular responses to the environment. TFs are classified into structural families based on previously described DNA-binding domains (DBDs) (Weirauch and Hughes, 2011). In eukaryotes, over 80 families of TFs are recognized, and the list includes homeodomains (e.g. Hox genes), nuclear receptors (ESR1; estrogen receptor) and P53 (TP53; tumor suppressor). Additional families of TFs are believed to exist (Weirauch and Hughes, 2011). The structure of a TF contains at least one DBD. However, they can have more than one DBD and additional regulatory domains (Frietze and Farnham, 2011). Therefore, TFs and their regulation have a global effect on cellular phenotypes, highlighting the need for their identification. Advances in DNA sequencing technologies have greatly increased the number of available genome sequences, enabling researchers to interrogate genetic and molecular mechanisms in their favorite systems (Hotaling *et al.*, 2021). A typical goal is to unravel gene regulatory networks controlling development in a biological system, and a common first step is to identify which TFs are expressed in specific tissues. A comprehensive catalog of all TFs encoded by a genome is oftentimes missing, especially in non-model organisms. With an ever-increasing number of available genomes, there is a need for tools that can aid downstream characterization and make the most of these genomes. To address this need, we present the Cis-regulatory Element-binding Protein Elucidator (CREPE), a tool to catalog and annotate TF proteins.

## 2 Description

CREPE is a pipeline designed to automate TF cataloguing and annotation through an interactive user interface. It is designed to perform two main functions: TF (i) cataloguing and (ii) annotation.

TF cataloguing: TFs are classified into structural families according to their DBDs. By parsing genome assemblies for previously described DBDs, it is possible to identify putative TFs. To run the TF Cataloguing function requires a species name and a protein sequence file (.fasta). A protein domain search analysis is performed using hmmscan from the HMMER Suite (Eddy, 2011) to scan the protein sequences against 82 curated eukaryotic TF DBD models (Weirauch and Hughes, 2011) (.hmm) from PFAM (Mistry *et al.*, 2021). Next, CREPE displays a TF family distribution plot and provides a sequence file with the identified putative TFs (CREPE_FL.fasta). This file can now be used for phylogenetic inferences using the researcher’s software of choice.

TF annotation: To run the TF Annotation function requires gene trees (in Newick format) of the putative TFs previously run by the user, and a metadata tab-separated file (.tsv) which cross-references a sequence id to its gene symbol. During execution, each tree is parsed to assign the putative TF the gene symbol of its closest relative in the gene tree as measured by the patristic distance using the ape 5.0 package (Paradis and Schliep, 2019). After execution, CREPE displays a table showing the resulting mappings of the putative TFs and makes them available for download.

Custom analysis: CREPE is designed to streamline TF identification but is limited by the TF DBD models used for searching. Nevertheless, we recognize that users may want to run custom TF cataloguing analysis using alternate tools or parameters. We created the Custom Analysis function to allow users to use CREPE to parse and visualize alternative analysis. Instructions on input formatting and execution for this function are available in the GitHub.

## 3 Benchmark

**Fig. 1.**
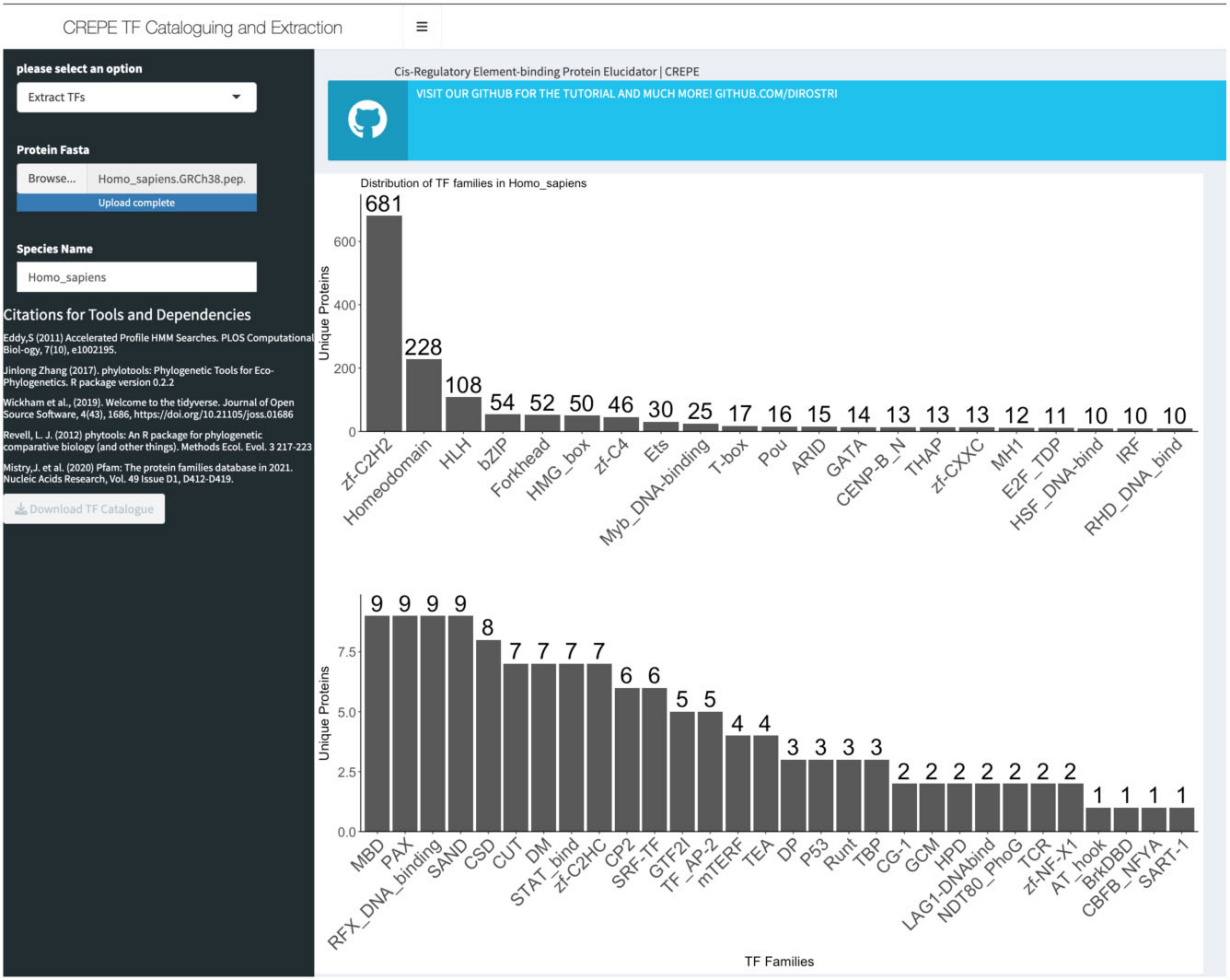
CREPE output after running the TF catalogue function. The main panel shows the distribution of TFs by family. In this example, we are using a non-redundant human proteome of 1519 human TFs

We benchmarked CREPE’s performance to manually curated human TF datasets. We applied the TF Cataloguing function of CREPE to the human proteome (GRCh38.p13, Ensembl 107) (Cunningham *et al.*, 2022). The proteome was pre-processed to only include the primary isoform per gene [from 120 712 to 23 486 sequences], and sequences derived from alternative mappings were removed [from 23 486 to 20 409 sequences]. We identified 1519 genes (∼7.4% of the input sequences) as putative TFs across 51 families (Supplementary Table S1). As expected, the five largest TF families were the C2H2 zinc fingers (zf-C2H2), homeodomains, basic helix–loop–helix (bHLH), basic leucine zippers (bZIP) and forkhead (Fig. 1).

**Fig. 2.**
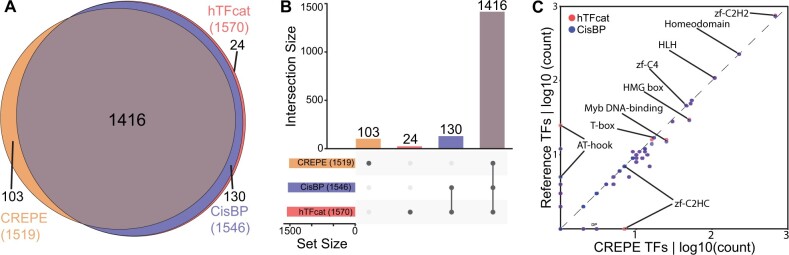
CREPE benchmarking. (**A**) Venn diagram and (**B**) upset plot showing overlap between the CREPE TF catalog, CisBP and hTFcat. (**C**) Scatter plot of TFs identified by CREPE with TFs from CisBP and hTFcat by TF family; dashed line shows 1:1 relationship

To validate our findings, we compared them to curated human TF datasets: CisBP 2.00 (Weirauch *et al.*, 2014), which was pre-processed to include TFs with known TF DBD [from 1639 to 1546 genes] and the Human Transcription Factors catalog (hTFcat) (Lambert *et al.*, 2018), which was also pre-processed to remove TFs with unknown DBDs [from 1639 to 1570 genes]. CREPE retrieved 91.6% (1416/1546) of the TFs in CisBP and 90.2% (1416/1570) of the TFs in the hTFcat (Fig. 2A and B). Comparison by family shows that 28 of the 51 (55%) TF families identified by CREPE showed parity (Fig. 2C;Supplementary Table S2).

CREPE identified 103 genes as TFs across 21 families, which were not included in the reference TF datasets (Supplementary Fig. S1A and Supplementary Table S2). The largest family included in this group (21/103; 20%) are the high-mobility group box, and includes HMGB1-4, TOX and TOX2-4 which to date, have not yielded DNA-binding specificity motifs (Lambert *et al.*, 2018). The second largest family is the C2H2 zinc fingers with 20 genes, of which 11 are annotated by Ensembl as novel and 3 as read-throughs (Cunningham *et al.*, 2022) (Supplementary Fig. S1B and Supplementary Table S2). To understand the generalities of the CREPE-only group, we performed Gene Ontology (The Gene Ontology Consortium, 2021) analysis and found overrepresentation of epigenetic terms, such as histone H3-K4 demethylation (GO:0034720), histone H3-K9 trimethylation (GO:0036124) and histone H4-K20 methylation (GO:0034770) (Supplementary Table S3), and contains genes such KDM5A, ARID4B, NSD2 whose debate of whether they are sequence specific TFs is ongoing (Lambert *et al.*, 2018). However, this group also contains genes such as HSFX3, HSFX4, DUXB, FOXL3, KLF18 that are members of known TF families. Whether they possess any DNA-binding sequence-specificity and TF activity needs to be validated experimentally.

CREPE missed 130 genes that are in both reference human TF datasets, spanning 16 families (Supplementary Fig. S2). The largest family in this group accounting for over half of the genes (79/130; 60.8%) are C2H2 zinc fingers. A notable difference between CREPE and the references appeared in the AT Hook and C2H2 zinc finger families. This was expected because it is known that current models can make the classification of C2H2 zinc fingers and AT Hook DBDs difficult (Lambert *et al.*, 2018). Taken together, our strategy using CREPE to identify the human TFs is summarized with a true positive rate of 90.2%.

## 4 Application

We applied CREPE to catalog the TF repertoire of *Heliconius melpomene* and *Heliconius erato*, two species of butterflies that are found throughout Central and South America and are a popular model system to study phenotypic determination through their wing coloration patterning mimicry (McMillan *et al.*, 2020). We obtained predicted proteomes for both species from LepBase (Challi *et al.*, 2016). Next, we applied the CREPE TF Cataloguing function and catalogued 664 and 599 TFs in *H.melpomene* and *H.erato*, respectively (Supplementary Fig. S3). In both species, TFs were distributed across 51 families. The top three families in both butterflies are zf-C2H2, homeodomain and bHLH, accounting for over half of the identified putative TFs. In preparation to run the CREPE TF Annotation function, we generated the required putative TF gene trees using OrthoFinder (Emms and Kelly, 2019) and 20 animal proteomes from Ensemble (Cunningham *et al.*, 2022) (Supplementary Table S4). These gene trees were then used as inputs for the TF Annotation function. The annotations of the putative TFs for *H.erato* and *H.melpomene* mapped to *Drosophila melanogaster* are available in our GitHub.

## 5 Conclusion

In this work, we described CREPE, a Shiny app to systematically catalogue and annotate TFs. CREPE was benchmarked against curated human TFs datasets, obtaining a 90.2% true positive rate. Using CREPE, we identified putative TFs that are currently not listed in curated references, suggesting that by using CREPE a user can obtain an updated TF catalog. The intention behind this tool is to allow researchers to explore the TFs in poorly annotated genomes or from understudied organisms. To showcase this functionality, we catalogued the TF repertoire of *H.melpomene* and *H.erato* butterflies. Taken together, CREPE provides a path forward for large-scale TF identification. A tutorial on how to execute CREPE and all data underlying this article can be found in our GitHub (github.com/dirostri/CREPE) and in its online supplementary material.

## Supplementary Material

vbad055_Supplementary_Data
